# Prooxidant/Antioxidant Ratio (ProAntidex) as a Better Index of Net Free Radical Scavenging Potential

**DOI:** 10.3390/molecules15117884

**Published:** 2010-11-03

**Authors:** Lai Teng Ling, Uma D Palanisamy, Hwee Ming Cheng

**Affiliations:** 1 Department of Physiology, Faculty of Medicine, 50603, University of Malaya, Kuala Lumpur, Malaysia; E-Mail: chenghm@um.edu.my (H.M.C.); 2 School of Medicine and Health Sciences, Monash University Sunway Campus, 46150, Petaling Jaya, Malaysia; E-Mail: umadevi.palanisamy@med.monash.edu.my (U.D.P.)

**Keywords:** pro-oxidant, DPPH, reducing power, antioxidants, Emblica^TM^

## Abstract

The antioxidant activity of several Malaysian plant extracts was analyzed simultaneously with their pro-oxidant capacity. This ratio represents an index (ProAntidex) of the net free radical scavenging ability of whole plant extracts. We observed that ethanolic extracts of *Nephelium lappaceum* peel, *Fragaria x ananassa* leaf, *Lawsonia inermis* leaf, *Syzygium aqueum* leaf and grape seed had a lower Pro-Antidex than the commercially available Emblica™ extract which is an antioxidant agent with very low pro-oxidant activity. Among the aqueous extracts, *Lawsonia inermis* leaf, *Nephelium mutobile* leaf and grape seed had lower pro-oxidant activity compared to the Emblica™ extract. Among these extracts, aqueous extract of *Nephelium mutobile* leaf had a very low index of 0.05 compared to 0.69 for Emblica™. Most of the extracts had a far lower ProAntidex compared to the Vitamin C. The index enables us to identify extracts with high net free radical scavenging activity potential. The ProAntidex is beneficial as a screening parameter to the food industries and healthcare.

## 1. Introduction

Antioxidants are substances that protect other chemicals in the body from damaging oxidation reactions by reacting with free radicals and other reactive oxygen species within the body, hence hindering the process of oxidation [1]. Plants contain active components, namely phenolics and polyphenolics, that are known to act as antioxidants [2]. Every antioxidant is in fact a redox agent and might become a pro-oxidant to accelerate lipid peroxidation and induce DNA damage under special conditions and concentrations [3]. Studies have revealed pro-oxidant effects of antioxidant vitamins and several classes of plant-derived polyphenols such as flavonoids [4], tannins [5] and curcumin [6]. As reported earlier, resveratol [3] and phloroglucinols from *Garcinia subelliptica *[7] can exhibit pro-oxidant properties, leading to oxidative breakage of cellular DNA in the presence of transition metal ions such as copper. Therefore, it is essential to discover natural compounds that have high net antioxidant activity.

The pro-oxidant and antioxidant effect of plant extracts are due to the balance of two activities: free radical-scavenging activity and reducing power on iron ions, which may drive the Fenton reaction via reduction of iron ions. In a Fenton reaction, Fe^2+^ reacts with H_2_O_2_, resulting in the production of hydroxyl radicals, which are considered to be the most harmful radicals to biomolecules. Fe^2+^ is initially oxidized to Fe^3+^ in the Fenton reaction. By the action of many reductants, such as ascorbic acid, the oxidized forms of iron ion can be reduced later to the reduced form (Fe^2+^), which can enhance the generation of hydroxyl radicals. A predominant reducing power (on iron ions) over the free radical-scavenging activity in a mixture of compounds results in the pro-oxidant effect [8]. In this study, the pro-oxidant capacity of the extracts were compared to the IC_50_ (mg/mL) of the antioxidant scavenging activity of DPPH radical. This ratio of pro-oxidant/antioxidant activity enabled us to evaluate the net antioxidant capacity of the extracts as this index will include not only the effective free radical-scavenging ability, taking into account pro-oxidant effect of the extracts. 

## 2. Results and Discussion

Most free radicals in biological systems are derivatives of oxygen (Reactive Oxygen Species, ROS), but there are also derivatives of nitrogen (Reactive Nitrogen Species, RNS). Conditions of high pro-oxidant activity due to free radicals are often described by the phrase oxidative stress. However, high concentrations of antioxidants may also have pro-oxidant activity. This can be best seen in the case of ascorbic acid, which is a well known pro-oxidant at very high concentrations [9]. This occurs as a result of the predominance of reducing power over its free radical scavenging activity. Consequently, ascorbic acid intake in individuals with high iron, especially in some premature infants, could be deleterious because it may cause oxidative damage to susceptible biomolecules [10,11,12].

Table 1 and Table 2 show the DPPH scavenging acitivity, pro-oxidant acitivity at an absorbance set at an arbitrary value of 1.0 and also the Pro-Antidex of the extracts. The lower ProAntidex values indicate that the extract had lower pro-oxidant activity, but high DPPH scavenging activity. Ethanolic extracts of each plants (*Nephelium lappaceum* peel, *Fragaria x ananassa* leaf, *Lawsonia inermis* leaf, *Syzygium aqueum* leaf and grape seed) had lower Pro-Antidex values than the commercially available Emblica™ extract (Table 1). Emblica^TM^, a well known commercial antioxidant on the market extracted from *Phyllanthus emblica*, is used as a comparison with the plant extracts [13]. The long-term antioxidant activity of Emblica^TM^ is based on a combination of low molecular weight tannins that form a cascading system of antioxidants. The key active ingredients of this cascade system are Emblicanin A and Emblicanin B. Under oxidation, Emblicanin A is transformed into Emblicanin B. Under continued oxidation, Emblicanin B then forms oligomers which themselves act as antioxidants [14]. We can observe the same trend for aqueous extracts. The aqueous extracts of each plant (*Lawsonia inermis* leaf, *Nephelium mutobile* leaf and grape seed) had lower Pro-Antidex values compared Emblica^TM ^(Table 2). It can be seen that all the extracts showed index values less than 0.7. These extracts possesed high DPPH scavenging activity but had low pro-oxidant activity. The ethanolic and aqueous extracts of grape seed showed an index value of 0.59. Aqueous extract of *Nephelium mutobile* leaf had ProAntidex of 0.05, which is far lower than that of Emblica^TM^, 0.6. Generally, all the plant extracts tested gave comparable results to Emblica^TM^ and far lower than Vitamin C. These extracts are potential nutraceutical antioxidants as ideally, such nutraceuticals should have good free radical scavenging ability and low pro-oxidant capacity. 

**Table 1:** DPPH scavenging activity, Pro-oxidant activity and ProAntidex in ethanolic extracts of selected Malaysian plants and standard. ProAntidex was devised using the ratio of pro-oxidant activities to the IC_50 _DPPH scavenging activity. All values represent means ± SD, *n *= 3.

Ethanolic Extract	Plant Part	DPPH (IC_50_, mg/mL)	Pro-oxidant^a^(mg/mL)	Pro-Antidex
*Azadirachta indica *	leaf	0.74 ± 0.46	0.50 ± 0.02	0.91 ± 0.58
*Mangifera indica*	leaf	0.17 ± 0.02	0.22 ± 0.03	1.32 ± 0.29
*Garcinia mangostana*	peel	0.11 ± 0.02	0.17 ± 0.04	1.62 ± 0.40
***Nephelium lappaceum***	**peel**	**0.12 ± 0.05**	**0.05 ± 0.04**	**0.48 ± 0.37**
*Psidium guajava*	leaf	0.18 ± 0.08	0.20 ± 0.03	1.27 ± 0.60
***Fragaria x ananassa***	**leaf**	**1.87 ± 0.80**	**0.99 ± 0.34**	**0.64 ± 0.45**
***Lawsonia inermis***	**leaf**	**1.3 ± 0.18**	**0.54 ± 0.07**	**0.43±0.11**
***Syzygium aqueum***	**leaf**	**0.22 ± 0.02**	**0.13 ± 0.03**	**0.62 ± 0.13**
*Nephelium lappaceum*	leaf	0.33 ± 0.03	0.76 ± 0.42	2.37 ± 1.40
*Peltophorum pterocarpum*	leaf	0.17 ± 0.12	0.11 ± 0.03	0.83 ± 0.44
*Peltophorum pterocarpum*	bark	0.10 ± 0.04	0.08 ± 0.03	0.94 ± 0.66
*Artocarpus champeden*	leaf	0.30 ± 0.21	0.75 ± 0.51	3.82 ± 4.24
*Nephelium mutobile*	leaf	0.24 ± 0.03	0.18 ± 0.06	0.76 ± 0.28
***Vitis vinifera***	**seed**	**0.15 ± 0.10**	**0.07 ± 0.02**	**0.59 ± 0.33**

^a^ The pro-oxidant activities were calculated by linear regression of plots where x-axis represented the various concentrations (0.1 mg/mL–6 mg/mL) of test plant extracts while the y-axis represented the absorbance of the test plant extracts.

**Table 2:** DPPH scavenging activity, pro-oxidant and ProAntidex in aqueous extracts of selected Malaysian plants and standards. ProAntidex was devised using the ratio of pro-oxidant activities to the IC_50 _DPPH scavenging activity. All values represent means ± SD, *n *= 3. **Designates a significance difference from Emblica^TM^, *p *< 0.01.

Aqueous Extract	Plant Part	DPPH (IC_50_, mg/mL)	Pro-oxidant^a ^(mg/mL)	Pro-Antidex
*Azadirachta indica *	leaf	0.96 ± 0.14	1.13 ± 0.01	1.20 ± 0.18
*Mangifera indica*	leaf	0.49 ± 0.39	1.03 ± 0.88	2.03 ± 1.38
*Garcinia mangostana*	peel	1.66 ± 2.4	3.04 ± 0.08	7.26±6.12**
***Nephelium lappaceum***	**peel**	0.54 ± 0.15	0.55 ± 0.37	1.08 ± 0.65
*Psidium guajava*	leaf	0.22 ± 0.01	0.42 ± 0.33	1.89 ± 1.43
***Fragaria x ananassa***	**leaf**	0.37 ± 0.07	0.58 ± 0.003	1.60 ± 0.29
***Lawsonia inermis***	**leaf**	**3.71 ± 0.34**	**0.38 ± 0.06**	**0.43±0.11**
***Syzygium aqueum***	**leaf**	0.33 ± 0.07	0.26 ± 0.09	0.88 ± 0.51
*Nephelium lappaceum*	leaf	0.67 ± 0.02	>2	2.37±1.40
*Peltophorum pterocarpum*	leaf	0.16 ± 0.05	0.17 ± 0.01	1.14 ± 0.45
*Peltophorum pterocarpum*	bark	0.20 ± 0.12	0.23 ± 0.01	1.48 ± 0.88
*Artocarpus champeden*	leaf	0.22 ± 0.01	0.20 ± 0.001	0.93 ± 0.05
*Nephelium mutobile*	leaf	**3.76 ± 0.27**	**0.18 ± 0.01**	**0.05 ± 0.01**
***Vitis vinifera***	**seed**	**0.46 ± 0.18**	**0.24 ± 0.11**	**0.59 ± 0.35**
*Green tea*	NA	0.28 ± 0.04	0.23 ± 0.07	0.82 ± 0.28
*Emblica*™	NA	0.31 ± 0.07	0.27 ± 0.12	0.69 ± 0.18
*Vitamin C*	NA	0.01 ± 0.00	0.03 ± 0.35	4.10 ± 3.36

^a^ The pro-oxidant activities were calculated by linear regression of plots where x-axis represented the various concentrations (0.1 mg/mL–6 mg/mL) of test plant extracts while the y-axis represented the absorbance of the test plant extracts.

**Figure 1:**
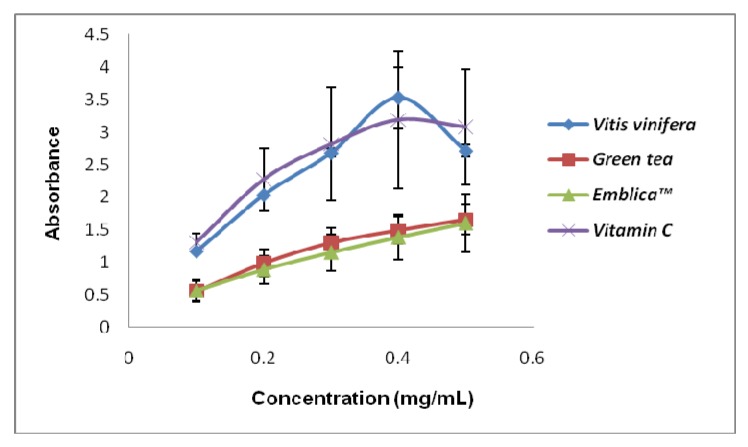
Pro-oxidant activity of the standards used in this study. The pro-oxidant assay was done by measuring reducing power on Fe^3+^ in the Fenton reaction. *Vitis vinifera *seed (grape seed), green tea, Emblica^TM^ and Vitamin C are positive controls that were used in this study for comparisons. All values represent means ± SD, *n *= 3.

**Figure 2:**
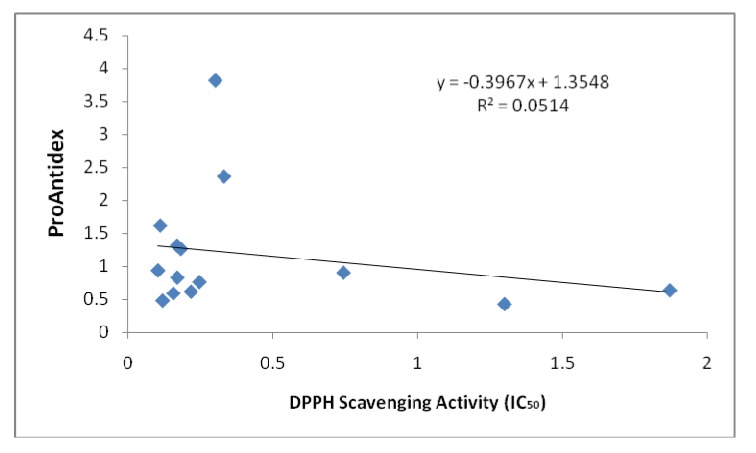
Correlation between ProAntidex and DPPH scavenging activity of ethanolic extracts that we used in this study.

**Figure 3:**
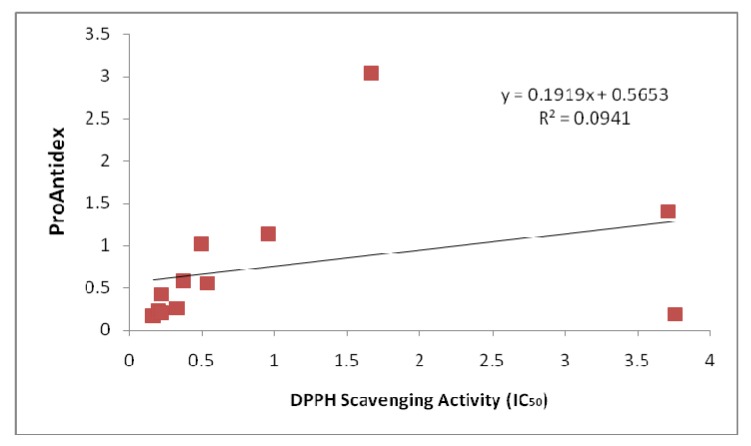
Correlation between ProAntidex and DPPH scavenging activity of aqueous extracts that we used in this study.

The main idea of this study is to use plant extracts as the ingredient in the formulation of a new antioxidant nutraceutical. Therefore, it is crucial for us to compare our extracts with both the natural and synthetic sources that are available. Natural plant extracts would be good alternative antioxidant sources in addition to the synthetic antioxidants [15,16]. Figure 1 showed the pro-oxidant activity of the standards that we used to compare with the plant extracts. Green tea and Emblica^TM ^possessed very low Fe ^3+ ^reducing power compared to grape seed and vitamin C. We can observe that the pro-oxidant activity of the standards increased proportionally with the concentrations used. The pro-oxidant and antioxidant effect of plant extracts are due to the balance of two activities, free radical-scavenging activity and pro-oxidant, reducing power on iron ions, which may drive the Fenton reaction via reduction of iron ions. Generally there is relationship between antioxidative and prooxidative activities and oxidation potentials [17]. The predominance of reducing power (on iron ions) over the free radical-scavenging activity results in the pro-oxidant effect [8]. The net antioxidant ability measured as as the ProAntidex can be used to screen the plant extracts, to see the balance between the free radical scavenging activity and the pro-oxidant capacity. The predominance of free radical scavenging activity over the reducing power of iron ions will result in net antioxidant effect of the whole extracts. On the other hand, if the reducing power of the extracts predominates, the free radical scavenging activity, pro-oxidant effect will result, even though the plant displayed high radical scavenging activity is only the antioxidant test. The significance of pro-oxidant activity is seen in the correlation of the Pro-Antidex and DPPH scavenging activity. Figure 2 and Figure 3 show the correlation analysis of the Pro-Antidex and DPPH scavenging acitivity. The correlation was very low (R^2 ^= 0.0514) for ethanolic extracts and R^2 ^= 0.0941 for aqueous extracts. There is no correlation between pure antioxidant activity and the net antioxidant potential (ProAntidex) in the whole plant extracts. Therefore, it is important for us to take into account the pro-oxidant activity together with the free radical scavenging activity of the extracts to determine their net free radical scavenging capability.

## 3. Experimental

### General

Fresh Malaysian plants were obtained from Klang Valley in Malaysia. The plants were authenticated by a botanist at the Herbarium of the Forest Research Institute of Malaysia (FRIM, Kepong, Malaysia). The selected plants in this study were *Azadirachta indica *leaf, *Mangifera indica *leaf, *Garcinia mangostana *peel, *Nephelium lappaceum* leaf and peel, *Psidium guajava* leaf, *Fragaria x ananassa* leaf, *Lawsonia inermis* leaf, *Syzygium aqueum* leaf, *Peltophorum pterocarpum* leaf and bark, *Artocarpus champeden *leaf and *Nephelium mutobile* leaf. The standards used in this study were vitamin C, green tea, Emblica^TM^ and *Vitis vinifera *seed. 

The leaves were washed with copious amounts of water followed by distilled water and then allowed to air dry at room temperature. They were then placed in an oven at 40 °C until completely dry, after which they were pulverized using a Waring blender or milled using a Fritsch dry miller. Water and solvent extraction: deionized water or the respective solvents (analytical grade) at 1:10 (w/v) concentrations were added to the powderized leaves. Water extraction was carried out at 40 °C while solvent extraction at room temperature for 24 hours in an orbital shaker. The suspension thus obtained was filtered using a 114 Whatman filter paper and filtrate collected. Aqueous filtrate was concentrated using a freeze drier while organic solvent filtrates were concentrated using a rotary evaporator. 

Scavenging activity on DPPH (1,1-diphenyl-2-picrylhydrazyl) free radicals by the extracts was assessed according to method reported previously [13]. A total of 950 µL of 0.004% DPPH ethanolic solution was added to 50 µL of an extract of different concentrations to make a final volume of 1 mL and the mixture was allowed to react at 37 °C. All determinations were performed in triplicate. After 10 minutes, the absorbance value was measured at 515 nm with Cary 50 Bio UV-visible spectrophotometer. The negative control used was 950 µL DPPH solution in ethanol with 50 µL of ethanol/distilled water while the positive controls used were L-ascorbic acid (vitamin C), green tea and grape seed.

Scavenging activity in this assay was expressed as IC_50_, which represents the concentration of the extract (mg/ml) required to inhibit 50% of the free radical scavenging activity. The optical density obtained was converted into of free radical scavenging activity by using the formula:

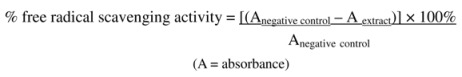



The IC_50_ values were calculated by linear regression of plots where x-axis represented the various concentrations (0.03125 mg/mL–5 mg/mL) of test plant extracts while the y-axis represented the average percentage of free radical scavenging activities from three replicates. The IC_50_ values of samples were compared against the standards, L-ascorbic acid and grape seed extract, and the lower the IC_50_ of a plant extract, the better it is as an antioxidant.

Reducing power of iron ion was measured according to the method of Ling *et al*. [18]. Equal volumes of the extract and 1% potassium ferricyanate [K_3_Fe(CN_6_)] were incubated at 50 °C for 20 minutes. An equal volume of 10% trichloroacetic acid was then added and mixture centrifuged at 3,000 g for 10 minutes. The upper layer of the solution (1.0 mL) was mixed with 1 mL of distilled water and 0.2 mL of 0.1% ferric chloride (FeCl_3_) and its absorbance recorded at 700 nm. Ethanol or distilled water was used as negative control while L-ascorbic acid, green tea, grape seed and Emblica^TM^ (a commercial antioxidant with very low pro-oxidant activity) was used as positive controls. 

The pro-oxidant activities were calculated by linear regression of plots where x-axis represented the various concentrations (0.1 mg/mL–6 mg/mL) of test plant extracts while the y-axis represented the absorbance of the test plant extracts. 

A net antioxidant potential was devised using the ratio of pro-oxidant activities at absorbance of 1.0 to the IC_50 _DPPH scavenging activity in both the total aqueous and ethanolic extracts. This ratio represents an index (ProAntidex) of the net free radical scavenging ability of whole plant extracts. The lower the ProAntidex parameter of the sample, the more efficient would be expected of the sample in neutralizing free radicals:

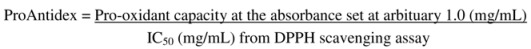



Each of the measurements described was carried out in three replicate experiments for different batches of the samples. Results are reported as mean ± standard deviation. Statistical analyses were performed by an analysis of variance (One-way ANOVA and nonparametric) using Dunnett tests for comparisons of samples with the control. A value of *P *< 0.05 was considered significant.

## 4. Conclusions

Pro-Antidex is a useful indicator in free radical research. The ratio of pro-oxidant to the antioxidant activity capacity gives a better picture of the real antioxidant capacity of plant extracts. The pro-oxidant assay will enable the nutritionists and chemists to formulate antioxidant mixtures that balance between the two activities, which is higher antioxidant activity with lower pro-oxidant capacity. In other words, the net ProAntidex should be low to reflect that the particular plant extract has good overriding antioxidant properties. 
